# Enhancement of the prediction of the openness of fresh-cut roses with an improved YOLOv8s model validated by an automatic Grading Machine

**DOI:** 10.3389/fpls.2025.1546503

**Published:** 2025-03-25

**Authors:** Qinghui Lai, Zhanwei Yang, Wei Su, Chuang Yan, Qinghui Zhao, Yu Tan, Yu Que, Jing Zheng

**Affiliations:** ^1^ School of Energy and Environmental Science, Yunnan Normal University, Kunming, China; ^2^ Faculty of Modern Agricultural Engineering, Kunming University of Science and Technology, Kunming, China

**Keywords:** YOLOv8, fresh-cut roses, openness grading, automatic grading machine, openness detection

## Abstract

**Introduction:**

The openness grading of fresh-cut roses relies heavily on manual work, which can be inefficient and inconsistent.

**Methods:**

In this study, an improved YOLOv8s model is proposed for openness grading in conjunction with a newly developed automatic grading machine for fresh-cut roses. The model identifies unopened inner petals and classifies openness into five levels: degree 1, degree 2, degree 3, degree 4, and deformity. To enhance detection accuracy while reducing the model complexity and computation, the backbone network of YOLOv8s is replaced by MobileNetV3. Additionally, an Efficient Multi-scale Attention (EMA) module is introduced to enhance focus on critical features, and a Wise-IoU loss function is incorporated to accelerate convergence.

**Results:**

Field experiments revealed that the openness predictions made by the automatic fresh-cut roses grader had errors of 6.9%, 9.1%, 10.0%, 6.5%, and 12.6%, respectively, compared to manual predictions.

**Discussion:**

Therefore, the improved YOLOv8s-F model effectively meets the requirements of fresh-cut rose openness grading.

## 1 Introduction

As one of the most popular fresh-cut flower varieties, roses play a significant role in flower cultivation and production. The production process includes harvesting, grading, packaging, and refrigeration. Openness is a crucial quality grading standard, as the accuracy of openness detection directly affects the consistency of fresh-cut roses after packaging, ultimately impacting their commercial value. Currently, openness grading predominantly depends on manual work, which is not only inefficient and costly but also subject to practitioner bias (Cui et al., 2023). This manual approach is insufficient to meet the demands of large-scale fresh-cut flower production. Developing an automatic grading machine for fresh-cut roses can achieve automatic grading, improve production efficiency, and increase economic returns. A visual detection system is a critical component of the grading machine, responsible for predicting the openness grade of each fresh-cut flower and providing grading signals to the slave device. The accuracy and speed of the system directly affect the grading performance. Therefore, fast and precise automatic openness detection is essential for improving fresh-cut rose grading and addressing current industry challenges.

Flower detection methods are broadly classified into two categories: deep learning-based and traditional image processing-based methods. Traditional image processing methods mainly identify and classify flowers based on color, edge features, and texture. For instance, Soleimanipour et al. (2019) developed an image-processing framework focused on detecting the geometric attributes of anthurium flowers. Their algorithm fitted B-spline curves at various rotation angles and utilized first- and second-order derivatives to calculate curvature and other key features by identifying crop boundaries. However, this approach is limited to 2D images and cannot capture advanced semantic features such as 3D attributes. Similarly, Feng et al. (2013) designed key components of an automatic transplanting machine for flower plug seedlings and assessed seedling growth using regional target pixel statistics. However, their algorithm lacked efficiency and robustness, as it did not focus on individual flower seedlings, leading to frequently missed detections, especially for smaller ones. Moreover, Aquino et al. (2015) proposed a method to segment grape inflorescence flowers in a field environment. Their algorithm applied region of interest (ROI) extraction and segmentation to HSV-format images, achieving 83.38% precision and 85.01% recall on 40 test images. However, as this method relied on pixel-by-pixel feature extraction, it required high computational power, making it unsuitable for edge device deployment and rapid detection. Furthermore, Sethy et al. (2019) introduced an approach for detecting and counting marigold flowers using HSV color transformation and circular Hough Transform (CHT), achieving a 5% detection error. However, this method requires high image quality when counting in open-field conditions, and its computational complexity limits its practicality for real-world production. Although the above traditional image processing approaches have solved some flower detection challenges, they require manual feature extraction operators, involve a high computational workload, and suffer from low computational efficiency.

Deep learning methods have demonstrated superior performance in detecting agricultural materials with high variability due to their robustness and ability to automatically extract semantic features—for instance, Tan et al. (2023) investigated Center Track, a video frame counting and tracking method for cotton seedlings and flowers, by improving CenterNet. Their method achieved AP50 = 0.962 on both seedling and flower datasets, with average relative errors of 5.5% and 10.8% for cotton seedling and flower count detection, respectively, compared to manual counting. However, the technique is limited to detecting and counting creamy-white immature flowers and cannot identify and analyze other maturity stages of cotton flowers. Moreover, Cıbuk et al. (2019) combined AlexNet and VGG16 for feature extraction and used the minimum redundancy maximum relevance (mRMR) method to select more effective features before classifying flowers with an SVM (support vector machine)–RBF (radial basis function) kernel. Their method achieved 95.7% mAP on the Flower102 dataset, focusing on flower species classification. However, it did not address maturity stage differentiation within a single flower species, limiting its application in plant growth stage monitoring. Furthermore, Anjani et al. (2021) employed the VGG16 deep convolutional neural network (CNN) algorithm with a dropout technique to classify rose flowers, achieving 96.33% mAP on the test set. While effective for low-resolution images (32 × 32 pixels), its performance decreases significantly for higher-resolution images, which may cause significant limitations in practical applications. In addition, Sun et al. (2021) introduced a rose openness classification method using InceptionV3, achieving 98% mAP by integrating image classification with bud depth information. However, the algorithm failed to account for the impact of packaging on detecting outer petal openness in fresh-cut roses, which could significantly affect the commercial value assessment of the classified flowers. In addition to that, Hui et al. (2023) improved the YOLOv5m model by inserting a convolutional block attention module (CBAM) for a safflower-picking robotic visual detection system. The improved model achieved a 5.5% increase in mAP, and in field harvest experiments, the safflower corolla picking rate exceeded 90% during the peak period. However, this approach significantly exceeded the metrics of the lightweight version of YOLOv5 in terms of memory requirements and computational overhead, making it unsuitable for edge computation deployment in field-picking robots. Furthermore, Wang Z. et al. (2022) optimized YOLO-v5 with a pruning algorithm to detect the apple stem/calyx position for pose detection. Their approach reduced the model size by 71% while retaining 88% of THE mAP. However, this technique is designed solely for ripe apples, excluding immature and green apples, limiting its application in full-cycle apple growth monitoring and quality assessment. Moreover, Wu et al. (2020) developed a channel-pruned YOLOv4 model for real-time apple blossom detection in natural environments. Their pruning approach reduced the model parameters by 96.74% and the model size by 231.51 MB, with only 0.24% mAP, providing a viable reference for apple flower thinning robots. While these studies have contributed to floral parameter extraction, target detection, classification, and counting, they do not directly apply to detecting openness grading in fresh-cut roses.

This study aims to develop an automatic grading machine for fresh-cut roses based on image processing. To achieve this goal, we designed an algorithm based on YOLOv8s to detect openness levels in fresh-cut roses. The key contributions of this study are as follows:

1. Development of an automatic grading machine with a simple structure and high efficiency for fresh-cut flowers.
2. Introduction of an improved YOLOv8s model, YOLOv8s-F, based on target detection method to achieve openness classification of fresh-cut roses.
3. Deployment of the YOLOv8s-F model to the grading machine for fresh-cut roses and comparison to manual grading.

## 2 Materials and methods

### 2.1 Overall structure and working principle of the visual classification system

**Figure 1:**
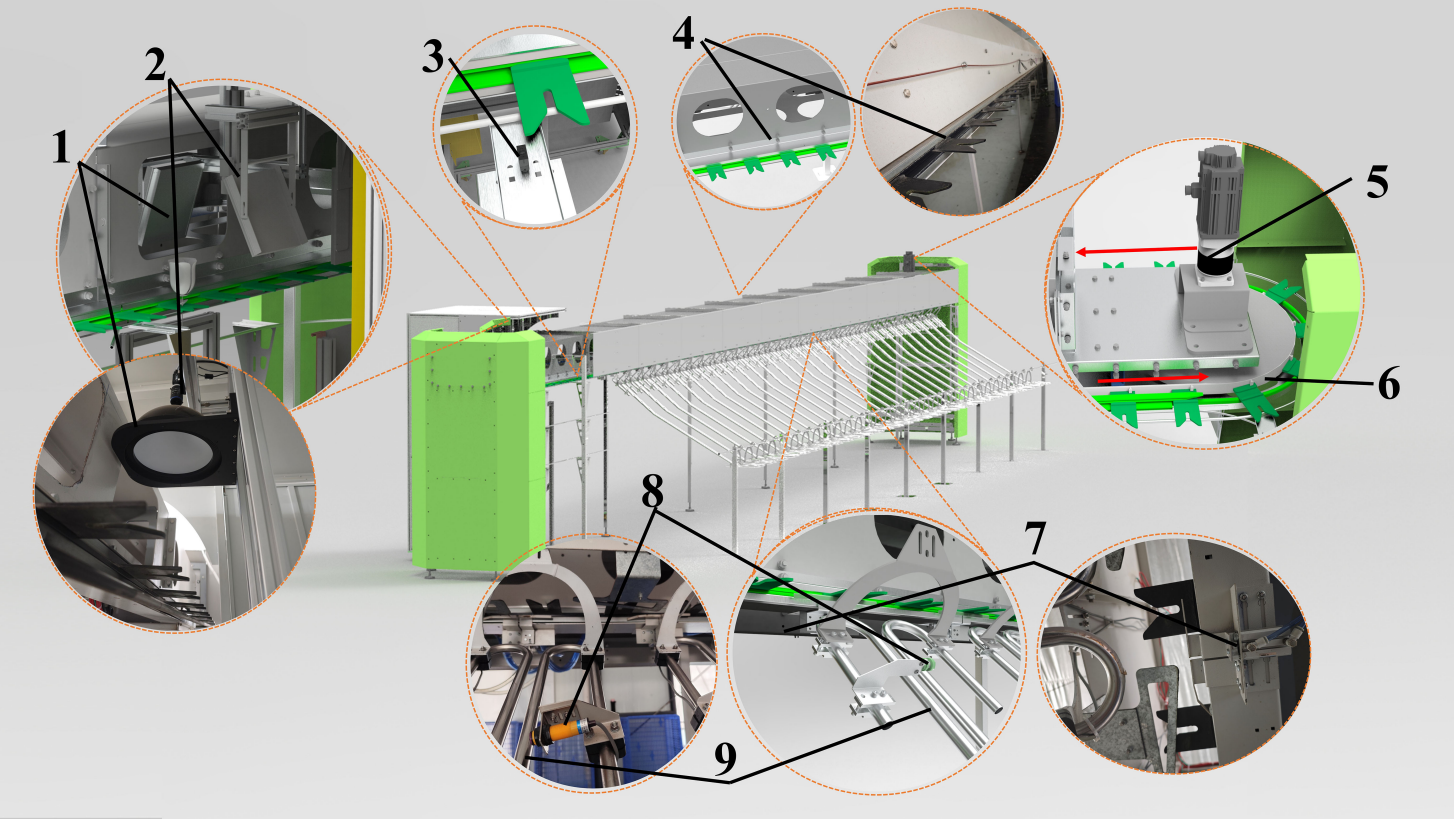
The whole machine includes (1) light source, (2) industrial camera, (3) compact photoelectric sensor, (4) flower-hanging device, (5) servo motor, (6) timing belt, (7) cylinders and solenoid valves and flower-pushing mechanism, (8) diffuse reflective sensors, and (9) flower-unloading device channel.

The main structure of the cut-flower visual grading device is illustrated in **Figure 1**. The machine is divided into four key components: transmission system, image acquisition system, flower-hanging device, and flower-unloading device. Specifically, this system comprises the following components:

- Light source: RY-MG300400 (OPT Machine Vision Tech Co., Ltd., Guangdong, China).
- Industrial camera: OPT-CC200-UM-0402.
- Compact photoelectric sensor: CX-442 (Panasonic, Osaka, Japan).
- Servo motor: ECMA-C20807RS (Delta Electronics (Dongguan) Co., Guangdong, China).
- Cylinders and solenoid valves: SDA50X10-B, 4V210-08 (Zhejiang Jorui Pneumatic Technology Co., Ltd., Zhejiang, China).
- Diffuse reflective sensors: E3F-DS10C4 (Shanghai DelixiSwitch Co.).

The host computer system consists of a PC, a programmable logic controller (PLC) (Siemens S7-1200 and its expansion module, Siemens PM207, SIEMENS AG, Germany), a switching power supply, and a servo controller (ASD-132-0721-B, Delta Electronics (Dongguan) Limited, Guangdong, China). The overall workflow of the system is defined as follows: after the user initiates the opening instruction via the PC, the control instruction is driven by the PLC and the servo motor controller, while the servo motor synchronizes the movement of the flower-hanging device.

**Figure 2:**
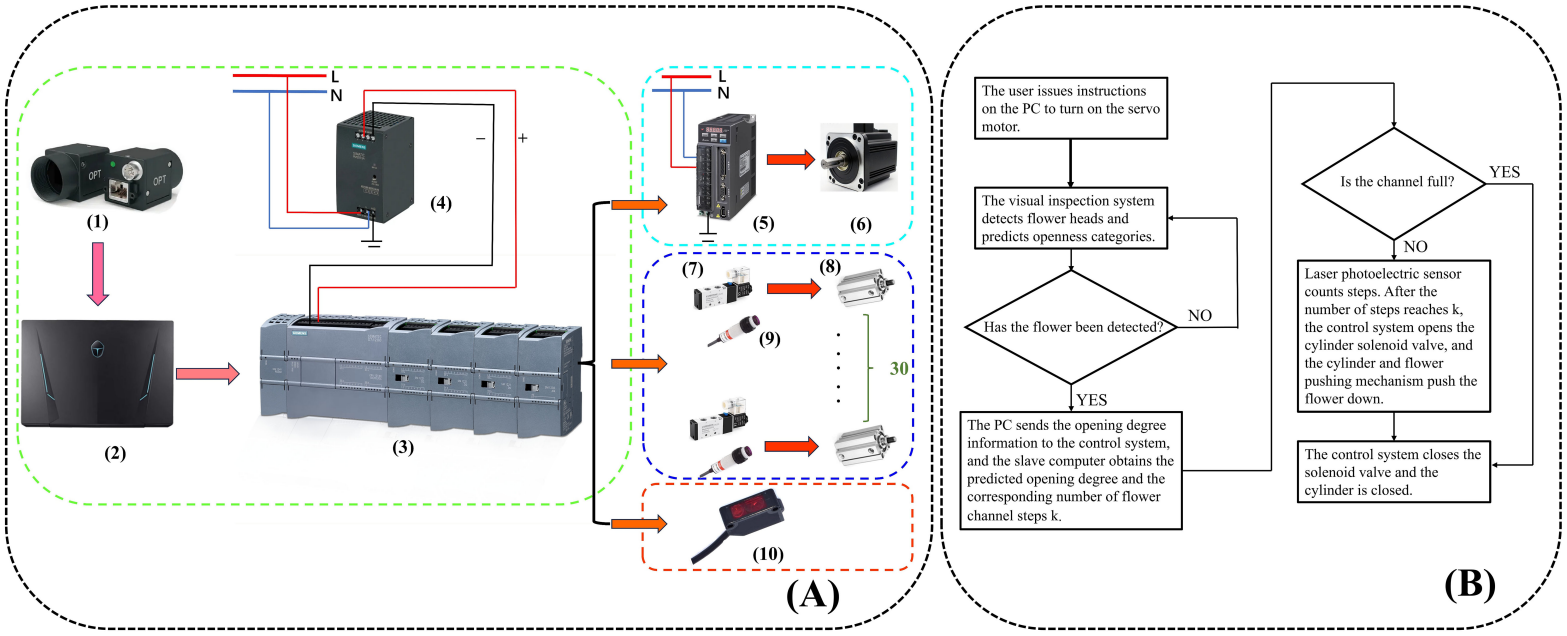
**(A)** Whole machine control flow chart: (1) industrial camera, (2) PC, (3) programmable logic controller (PLC), (4) switching power supply, (5) servo controller, (6) servo motor, (7) solenoid valves, (8) cylinders, (9) diffuse reflective sensors, and (10) compact photoelectric sensor. **(B)** Schematic diagram of the whole machine control.

As the flowers are placed in the flower-hanging device and transported by the synchronous belt, an industrial camera captures their images. The captured images are then transmitted to the PLC system after model interference is performed on the PC side. When a flower reaches the corresponding flower-pushing device, the PLC system controls the solenoid valve, triggering the cylinder to activate the flower-pushing device, which directs the fresh-cut flower to the designated unloading channel. In this workflow, the distance between adjacent flower-hanging devices is a fixed value. The compact photoelectric sensor (as shown in **Figure 1**) calculates the distance between the fresh-cut roses and the unloading channel by counting the number of flower-hanging devices. To prevent congestion, when an excessive accumulation of fresh-cut flowers in a single channel obstructs the diffuse sensor (**Figure 1**), the system temporarily suspends further flower placement in that channel. The control flowchart is presented in **Figure 2A**, while the schematic diagram of the control system is shown in **Figure 2B**.

### 2.2 Levels of openness

**Figure 3:**
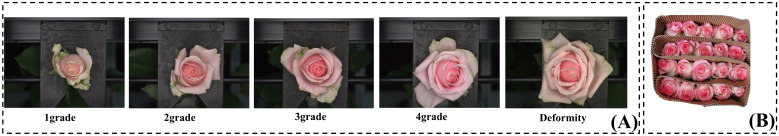
**(A)** Example of openness. **(B)** Example of packaging for fresh-cut roses.

Zhang (2017) encoded the openness of fresh-cut roses on a scale of 1 to 5 degrees. Guided by Zhang Li’s openness coding method, we modified the coding system to better align with actual production needs. Since fresh-cut roses with degrees 4 and 5 have limited storage and transport value in practical production, this study focuses on fresh-cut roses ranging from degree 1 to 3. To simplify the classification, we merged degrees 4 and 5 into a single category (degree 4). In addition, deformed roses, which lack commercial value, were categorized separately to prevent misclassification between degree 1 and deformed flowers. **Figure 3A** provides an example of the openness classification. According to GB/T 41201-2021 for the standard of the openness of Chinese roses belonging to Rosaceae, openness is primarily evaluated based on the position of the sepals, the degree of expansion of the inner and outer petals, and the degree of petal flipping. In the Kunming Dounan International Flowers Industrial Park (Yunnan Province, China), roses prepared for auction are usually packaged in corrugated cardboard boxes (**Figure 3B**) in bundles of 15–20. However, the packaging process affects the outer petals, sometimes causing an unnatural unfolding in the package. From a commercial perspective, practitioners prioritize the expansion and turning of the inner petals over the outer petals and sepals in the practical grading of fresh-cut roses. Therefore, we adjusted the openness grading standard to better reflect real-world production needs. **Table 1** shows the specific adjustments.

**Table 1:** Manual grading standard.

Openness	Standard
Degree 1	The outer petals are unopened or slightly open, and the center of the petal is pointed and has black spots.
Degree 2	The center of the petals opens, the petals begin to loosen, and the black dots disappear.
Degree 3	The inner petals are further loosened, the opening of the central part of the petals continues to increase, and the third petals are not separated.
Degree 4	The third layer of petals separates, and the multi-layer petals are turned outward.
Malformation	The third layer of petals is completely separated and turned over, the inner layer of petals is compact, and there is a black spot in the center of the petal.

### 2.3 Dataset collection and preprocessing

The image dataset for this study was collected from the National Flower Breeding Base in Luxi County, Honghe Autonomous Prefecture, Yunnan Province, China. Luxi County has a subtropical monsoon climate, with an average annual temperature of 15.5°C and an annual precipitation of 929 mm. The dataset was captured with an OPT 200 camera equipped with an FA lens. The camera lens was positioned 30 cm above the top of the flower.

We selected three main cultivars for this study: White Avalanche, Sweet Avalanche, and Peach Avalanche. The boundaries between adjacent openness levels in fresh-cut flowers under natural conditions are often blurred, and flower characteristics can vary within the same openness category. Additionally, the opening degree of fresh-cut roses in natural environments is affected by temperature; higher temperatures lead to greater flower opening, causing variations in the same openness level across different temperatures. To enhance image diversity, we collected 1,215 freshly cut roses at different growth stages during three time periods: morning, midday, and evening. To enrich the training set, improve feature extraction, and enhance the model’s generalization ability, we applied data augmentation techniques before network training. These included flipping, adding noise, optical distortion, scaling, and cropping. The augmentation parameters were set as follows: image flip ratio set to 0.5, noise addition ratio set to 0.3, zoom ratio set to 0.35, and crop ratio set to 0.4. After augmentation, 2,031 images were obtained in the final dataset. **Table 2** shows the number of categories. For annotation, we used *lableme* to label the tightly inner wrapped petals and their adjacent petal parts and saved them in JSON format. To ensure quality and accuracy, we conducted manual visual checking. Finally, the dataset was split into a training and a test set, respectively, with a ratio of 8:2.

**Table 2:** Number of categories in the dataset.

	Degree 1	Degree 2	Degree 3	Degree 4	Deformity
Avalanche	165	147	120	150	66
Sweet Avalanche	193	150	152	145	50
Peach Avalanche	150	143	172	138	90

### 2.4 Model structure

This section first outlines the strategy for data and model enhancement, followed by a description of the YOLOv8 baseline model and the proposed YOLOv8s-F model structure and its improvements. The model significantly enhances both the accuracy of fresh-cut flower openness detection and the detection speed of the process.

#### 2.4.1 YOLOV8 model

Building upon the classic single-stage object detection algorithm YOLO (Redmon et al., 2016), YOLOv8 achieves an optimal balance between speed and accuracy, making it widely adopted in both academia and industry. The model architecture consists of three parts: the backbone, the neck, and the head. The backbone is used for feature extraction, the neck is employed to fuse feature maps of different scales and connect the backbone to the head networks, and the head is used to predict bounding boxes and their corresponding labels.

Backbone and neck networks: YOLOv8 adopts the CSP-Darknet53 backbone from YOLOv3 (Redmon and Farhadi, 2018). Compared to YOLOv5 (Glenn, 2022), the C2f module with a multi-layer fusion residual structure provides a richer gradient flow while maintaining a lightweight design compared to the C3 module.

**Figure 4:**
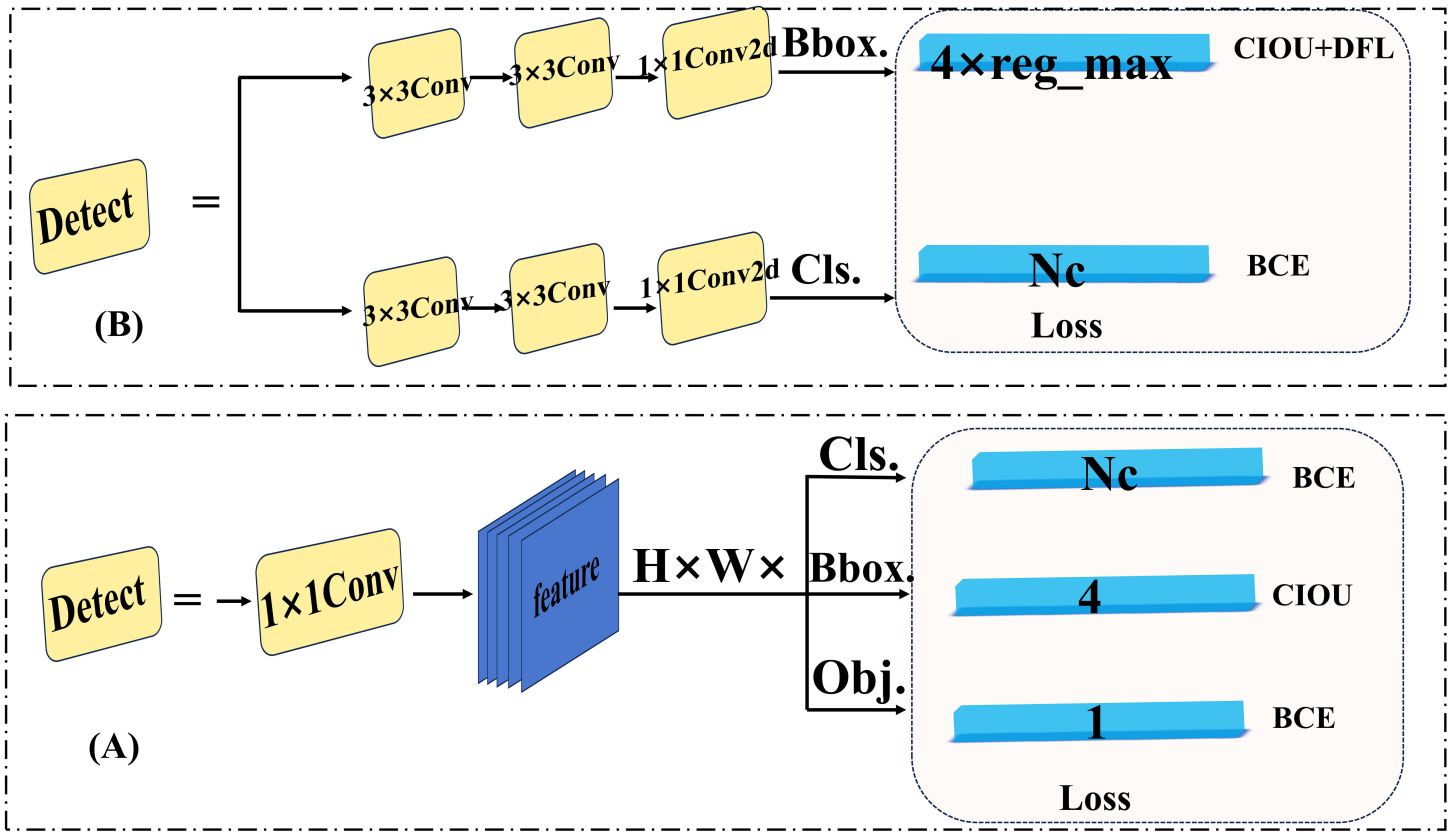
**(A)** YOLOv5 coupled head. **(B)** YOLOv8 decoupled head.

Head network: Unlike YOLOv5, which employs a coupled head, YOLOv8 adopts a decoupled head (Ge et al., 2021) (**Figure 4**). This separation of regression and classification tasks, along with independent loss calculations, enhances both network convergence speed and accuracy. Previous YOLO versions relied on anchor-based methods for allocating prediction boxes. However, these approaches required predefined hyperparameters (such as size and aspect ratio) that demanded heuristic tuning and dataset-specific adjustments, thus lacking generalizability (Zand et al., 2022). To address this, YOLOv8 employs an anchor-free allocation of prediction boxes, which increases the number of positive samples while significantly reducing the training parameters and computational complexity. Subsequent YOLO versions (YOLOv10) introduced the one-to-one head, eliminating the need for non-maximum suppression (NMS) during the inference phase. While this approach improves inference efficiency, it may compromise classification accuracy when distinguishing between highly similar objects. In summary, the model structure of YOLOv8 plays a crucial role in the multi-category detection of fresh-cut roses’ openness in this article.

Loss function: In the YOLOv8 model, the target detection task is performed using a combination of localization and classification losses, with the loss function being defined as follows (Equation 1):


(1) $$L=\lambda_{reg}L_{reg}+\lambda_{\text{cls}}L_{cls}$$

where $\lambda_{reg}$ and $\lambda_{cls}$ are hyperparameters that control the regression loss of the bounding boxes as well as the classification loss.

#### 2.4.2 Introduction to YOLOV8s-F

**Figure 5:**
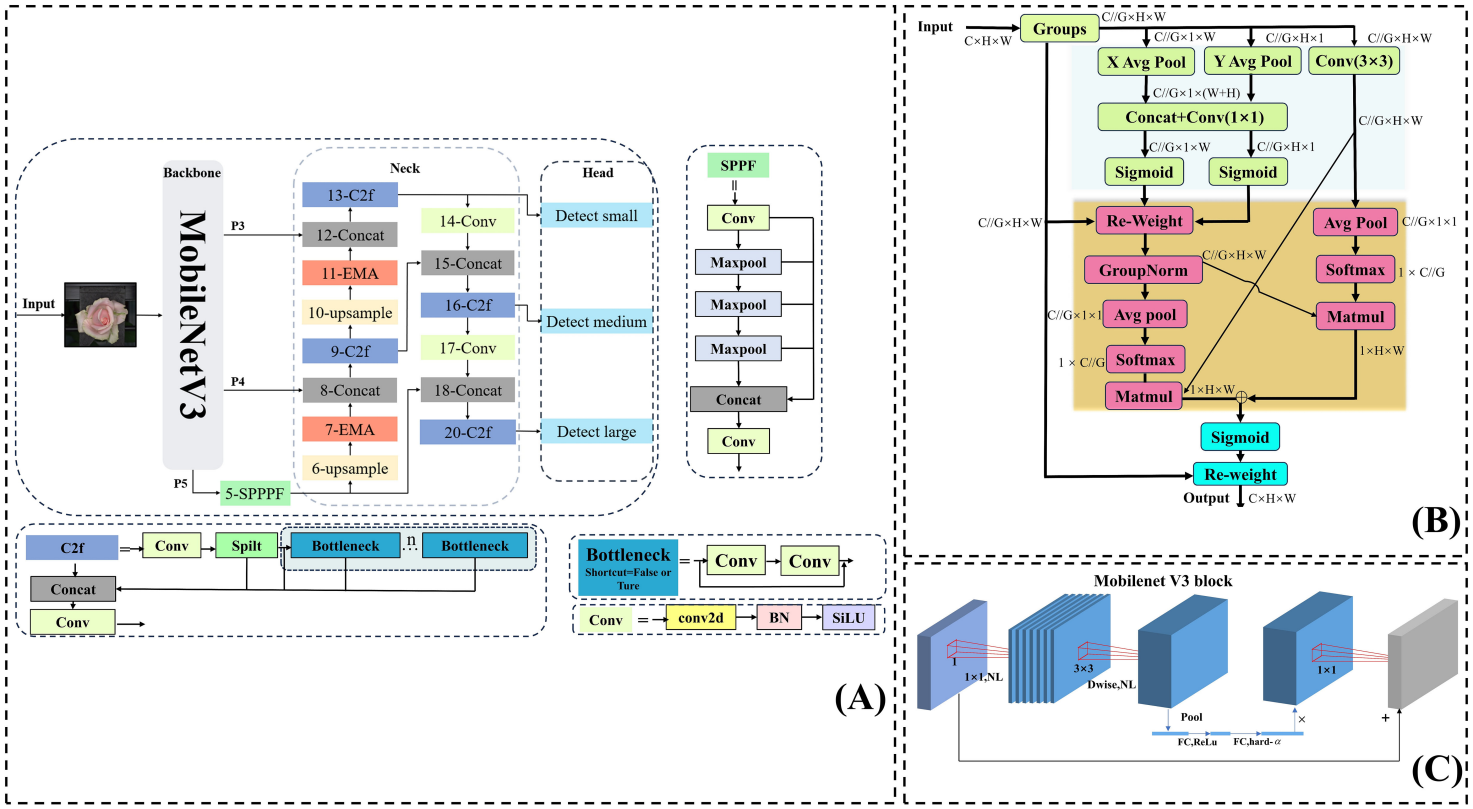
**(A)** Structure of the YOLOv8s-F model. **(B)** Structure of the EMA module. **(C)** Structure of the MobileNetV3 module.

The YOLOv8s-F model is a lightweight and efficient target detection model that has been specifically improved for detecting the openness of fresh-cut roses across different varieties and growing conditions. The specific improvements made to YOLOv8s are shown in **Figure 5A**:

1. We replaced the DarKnet53 backbone with the MobileNetV3 backbone (Howard et al., 2019). Moreover, we concatenated the feature maps of P4 and P3 with the upsampling steps in the neck network to reduce the computational load in the feature extraction part of the backbone while extracting rich details and semantic features.
2. We inserted the EMA module (Ouyang et al., 2023) twice before the concatenation process in the upsampling of the neck section, enabling the model to fully consider contextual semantic information while capturing global information in width and height directions.
3. We replaced the CIoU loss in the bounding box loss function with Wise-IoU (Tong et al., 2023), which dynamically adjusts its focus based on the target.

##### 2.4.2.1 MobileNetV3

MobileNetV3 builds upon the inverse residual structure introduced in MobileNetV2 (Sandler et al., 2018) (**Figure 5C**). Unlike traditional residual structures, which first reduce feature map dimensionality before enhancement, the inverse residual structure initially expands the dimensionality using a 1 × 1 lightweight dilated convolution, allowing for richer feature representation and extraction. Then, feature information is processed through depthwise separable convolutions composed of a 3 × 3 depth convolution and a 1 × 1 convolution, which separate spatial filtering from feature generation, maximizing the utilization of spatially corresponding features across different spaces while expanding to higher-dimensional feature spaces. Meanwhile, a 1 × 1 convolution can adjust the channel dimension to maintain a compact input–output structure. Mobilenetv3 also introduces an optional SE attention module within the residual, to calibrate channel-level feature responses through squeeze excitation operations (Hu et al., 2018). At present, MobileNetV3 has various applications in agricultural product defect detection, agricultural product testing, and agricultural product quality grading (Li et al., 2023; Wang L. et al., 2022; Zhu et al., 2023).

##### 2.4.2.2 EMA

Efficient Multi-scale Attention (EMA) represents an improvement over the CA attention mechanism, and its structure is shown in **Figure 5B**. For any input feature map, EMA is divided into channels. Grouping the input sub-feature maps avoids dimensionality reduction of the original feature map, significantly helping in preserving the semantic features of the original feature map. Similar to CA, the EMA’s 1 × 1 branch is encoded in the height and width directions by the (H,1) and (1,W) pooling kernels. Thus, for channel *C*, the outputs are computed after two parallel average pooling calculations. They are expressed as follows (Equations 2, 3):


(2) $$z_{c}^{H}\left(H\right)=\frac{1}{W}\sum_{0\leq i\leq W}x_{c}\left(H,i\right)$$

(3) $$z_{c}^{W}\left(W\right)=\frac{1}{H}\sum_{0\leq j\leq H}x_{c}\left(j,W\right)$$

EMA then performs a feature map transformation on $z_{c}^{W}$ and concatenates it within $z_{c}^{H}$ in the *H* dimension. Then, the split operation separates and re-aggregates with the input feature map. EMA additionally introduces a 3 × 3 branch containing a single 3 × 3 convolution to expand the feature space. Moreover, EMA proposes a method for aggregating information across space, where the encoded output of the 1 × 1 branch aggregation is multiplied by the output of the 3 × 3 branch, namely, ${\mathbb{R}}_{1}^{1\times C\text{//}G}\times {\mathbb{R}}_{3}^{C\text{//}G\times HW}$ . Similarly, the global spatial information encoded in the 3 × 3 branch is multiplied by the 1 × 1 branch, namely, ${\mathbb{R}}_{3}^{1\times C\text{//}G}\times {\mathbb{R}}_{1}^{C\text{//}G\times HW}$ . In addition, EMA aggregates channel attention in three directions to enhance feature representation. In the 1 × 1 branch, two sub-branches capture global information along two vertical directions and account for long distance dependencies in different orientations. Meanwhile, the 3 × 3 branch, with its larger receptive field, provides richer contextual information. The cross-space aggregation between the 1 × 1 and 3 × 3 branches ensures effective interaction between spatial features, allowing the model to integrate high-level semantic features in flowers.

##### 2.4.2.3 Wise-IoU

As a crucial component of visual models, the bounding box loss is a critical factor of the model. Traditional intersection over union (IoU) loss (Yu et al., 2016) calculates the difference between the IoU of the predicted bounding box and the ground truth, with a loss function defined as 1 - IoU. However, when the predicted and ground truth boxes do not intersect, the gradient becomes zero, leading to the gradient vanishing during backpropagation. To address this issue, Distance-IoU (DIoU) (Zheng et al., 2020) and Complete-IoU (CIoU) introduced penalty terms based on the distance between the center points of the predicted and ground truth boxes, as well as aspect ratio, incorporating them into the IoU loss function in an additive form. Wise-IoU further improves on this by constructing a distance attention $R_{WIoU}$ in a multiplicative form, introducing two layers of attention (as shown in **Equation 4**). When the prediction box overlaps well with the ground truth box, $R_{WIoU}$ based on $L_{IoU}$ will further reduce the gradient gain of that prediction box, preventing the model from focusing excessively on already well-aligned predictions. Conversely, when the prediction box coincides with the ground truth box, $R_{WIoU}$ will increase the gradient gain of that prediction box. Thus, the prediction box will have the highest gradient gain when its IoU reaches some specified value (Equation 4).


(4) $$L_{WIoUv1}=R_{WIoU}L_{IoU}$$

where $R_{WIoU}$ is the normalized distance between the prediction box and the centroid of the ground truth box, and $L_{IoU}$ represents the IoU loss between the ground truth and prediction boxes.

Based on $L_{WIoUv1}$ , $L_{WIoUv2}$ and $L_{WIoUv3}$ are constructed following a certain sequence to assign a smaller gradient gain to a high-quality target with a small outlier; thus, the model can focus on challenging targets and adjusts losses dynamically. In this way, the classification performance is improved as denoted below (Equations 5, 6):


(5) $$L_{WIoUv2}={\left(\beta \right)}^{\gamma }L_{WIoUv1}$$

(6) $$L_{WIoUv3}=(\frac{\beta }{\delta \alpha^{\beta -\delta }})L_{WIoUv1}$$

where *β* denotes the outlier describing the quality of the anchor box, whereas *σ* and *α* are hyperparameters.

### 2.5 Hyperparameters and evaluation indicators

In this study, the same dataset is employed to train both the YOLOv8s-F and YOLOv8s models. The PC has the following configuration: Windows operating system, CPU: i713700k, GPU: RTX4080, RAM: 32 GB, deep learning framework: Pytorch 2.1.0, development environment: Python 3.10. The training parameters are as follows: the input image size is set to 640 × 640 pixels, backpropagation uses stochastic gradient descent (SGD) to train the model, the batch size is set to 16, the initial learning rate is set to 0.01, and epoch is set to 300.

In this study, the performance of the YOLOv8s-F model is assessed based on the following evaluation metrics: precision (P) (**Equation 7**), recall (R) (**Equation 8**), mean average precision (mAP) (**Equation 9**), floating point operations per second (FLOPs), frames per second (FPS), and model size. The expressions are highlighted in **Equations 7**–**9**. Model size reflects the storage space of the model, FLOPs measure the complexity of the model, and FPS measure the speed of model inference.


(7) $$P=\frac{\text{TP}}{\text{TP}+\text{FP}}\times 100\%$$

(8) $$R=\frac{\text{TP}}{\text{TP}+\text{FN}}\times 100\%$$

(9) $$mAP=\frac{\int_{0}^{1}\text{P}(\text{R})\text{dR}}{\text{C}}$$

where TP represents the number of true positive samples predicted to be positive by the model, FP denotes the number of false positive samples predicted to be positive by the model, FN indicates the number of false negative samples predicted to be negative by the model, and *C* is the number of classes.

## 3 Experiment and results

### 3.1 Model training results

**Figure 6:**
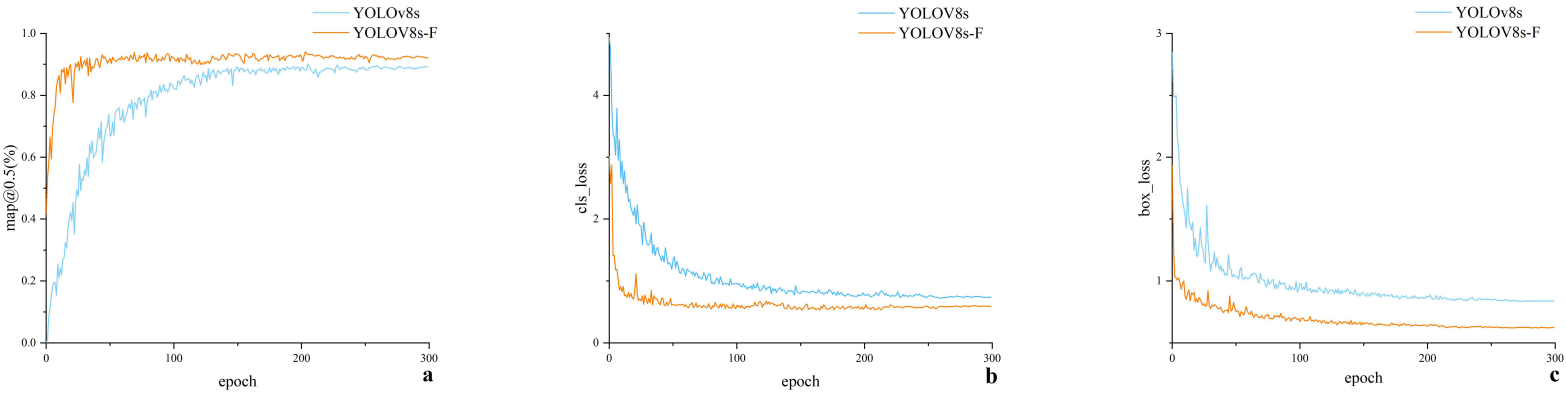
**(a-c)** Model training results.

In **Figure 6**, a represents the variation curve of mAP0.5@ (%) during training for both models, whereas *b* and *c* are the variation curves of bounding box loss and classification loss during training for both models. Moreover, in **Figure 6a**, the mAP@0.5 (%) values of both models experienced a rapid increase in the first 50 epochs, reaching a steady state after 200 epochs. Subsequently, the mAP@0.5 (%) values of both models showed minimal fluctuations, with YOLOv8s-F exhibiting significantly higher values compared to YOLOv8s. Referring to **Figures 6b, c**, the losses of both models in the first 50 epochs experienced a rapid decrease, and the bounding box loss values of these models reached a steady state after 200 epochs. As for the classification losses of the two models, they reached a steady state after 150 epochs. To sum up, YOLOv8s-F demonstrated faster convergence and lower loss values compared to YOLOv8s. An analysis of the experimental data suggests that the YOLOv8s-F model has superior training performance.

### 3.2 Ablation experiment

Comparative experiments were conducted on model performance using the same dataset and hyperparameters, and the results are displayed in **Table 3**. Introducing the MobileNetV3 backbone resulted in a 1.9% increase in mAP@0.5 (%), a 23.9% reduction in FLOPs, and a 31% decrease in model size, with improvements in precision and recall values. Moreover, the introduction of the EMA module led to a 1% increase in mAP@0.5 (%), with negligible impact on model size and FLOPs. In addition, we conducted experiments on the three versions of Wise-IoU, and the results indicated that Wise-IoUV3 had the most significant impact on model performance. Compared to the baseline model, it resulted in a 4.5% increase in mAP@0.5 (%), a 11% improvement in precision, a 16.5% reduction in FLOPs, and a 30% decrease in model size. The mAP@0.5 (%) findings for each category are shown in **Table 4**. These results demonstrate that our improvements to YOLOv8s significantly enhanced the model’s performance in detecting fresh-cut roses’ openness.

**Table 3:** Results of the ablation test.

Mobv3	EMA	Wiouv1	v2	v3	mAP@0.5 (%)	Precision (%)	Recall(%)	FLOPS(G)	Model size (MB)
					89.6	78.9	88.9	28.4	30.0
✓					91.5	87.7	87.9	21.6	20.7
✓	✓				92.5	87.1	86.7	23.7	20.8
✓	✓	✓			93.0	85.1	89.7	23.7	20.8
✓	✓		✓		93.0	85.1	89.7	23.7	20.8
✓	✓			✓	**94.1**	89.9	87.0	23.7	20.8

Bold font indicates mAP@0.5 %The maximum value.

**Table 4:** YOLOv8s-F mAP@0.5 (%) values for each category.

Category	AP@0.5 (%)
Degree 1	97.9
Degree 2	96.0
Degree 3	91.3
Degree 4	97.7
Deformity	87.6

### 3.3 Comparison of the YOLOv8s-F model with other latest models

YOLOV8s-F was also compared to SSD, Faster R-CNN, YOLOV3, YOLOV5s, YOLOV7, YOLOV8s, RT-DETR, and the updated versions of YOLOV10 and YOLOV11 models. All of these models were trained and tested on the same dataset, and the results are illustrated in **Table 5**. Based on the findings, Faster-RCNN achieved the highest mAP, precision, and recall with values of 95.0%, 93.8%, and 90.9%, respectively. However, with a FLOPs value of 269.18, it had the highest computational cost among the models and the lowest FPS at 37, highlighting a slower inference speed and a larger model size, which may not meet the requirements for rapid detection in practical production processes and may hinder deployment on lighter-weight devices subsequently.

**Table 5:** Results of the model comparison experiments.

Model	mAP@0.5 (%)	Precision (%)	Recall (%)	FLOPS (G)	Model size (MB)	FPS
SSD	81.2	80.9	83.0	32.58	104	55
Faster-Rcnn	95.0	93.8	90.9	269.18	315	37
YOLOv3	88.9	83.4	87.2	12.9	16.6	97
YOLOv5s	87.9	82.4	85.0	15.8	13.7	186
YOLOv7	88.2	78.9	85.3	105.2	71.3	170
YOLOv8s	89.6	78.9	88.9	28.4	30.0	174
YOLOv10s	89.0	80.1	83.3	30.1	29.4	177
YOLOv11s	88.5	79.4	86.1	29.5	30.1	172
Rt-DETR	89.3	80.2	84.4	110	66.1	139
Ours	94.1	89.9	87.2	23.7	20.8	188

Moreover, the proposed model achieved a mAP value of 94.1%, only 0.9% lower than Faster R-CNN, with FLOPs being only 8.8% compared to Faster R-CNN. Therefore, our model is more suitable for rapid detection requirements. Compared to SSD, YOLOv3, YOLOv5s, YOLOv7, YOLOv10s, YOLOv11s, RT-DETR, and YOLOv8s, our model improved mAP@0.5 (%) by 12.9%, 5.2%, 6.2%, 5.9%, 5.1%, 5.6%, 4.8%, and 4.5%, respectively. Our model’s precision and recall were 89.9% and 87.2%, slightly lower than those of Faster R-CNN, but it had the greatest advantage compared to other models. With a model size of 20.8 MB and 23.7 GFLOPs, slightly higher than YOLOv3 and YOLOv5s, our model maintained the highest FPS of 188 frames/s. Considering the model’s advantages in other metrics, the impact of the model size and GFLOPs on the model can be considered negligible.

### 3.4 Comparative experiment on attention mechanisms

Referring to **Table 6**, we conducted comparative experiments on YOLOv8s-F by adding EMA along with popular attention mechanisms: CA (Hou et al., 2021), CBAM (Woo et al., 2018), SE (Hu et al., 2018), and ECA (Wang et al., 2020). All attention mechanisms were implemented in the same location. The experimental results indicate that adding EMA achieves the highest mAP@0.5 (%) value of 94.1%, 1.8%, 0.6%, 2.3%, and 1% higher than CA, CBAM, SE, and ECA, respectively. Although adding EMA slightly increased the model size and FLOPs compared to other attention mechanisms, its impact on the actual performance of the model can be considered negligible. In addition, the experiment results show that the EMA attention mechanism is particularly well suited for detecting the openness of fresh-cut rose flowers. This can be attributed to EMA’s 1 × 1 cross-spatial semantic information aggregation branch and 3 × 3 branch, which enhance the model’s ability to capture long-distance dependencies and integrate channel information effectively. By leveraging these mechanisms, the EMA-enhanced model demonstrates superior performance.

**Table 6:** Comparative experiments on attention mechanisms.

	mAP@0.5 (%)	Model size (MB)	FLOPS (G)
+CA	92.3	20.2	21.7
+CBAM	93.5	20.8	21.3
+SE	91.8	20.2	21.6
+ECA	93.1	20.2	21.6
+EMA	94.1	20.8	23.7

### 3.5 Field experiment

**Figure 7:**
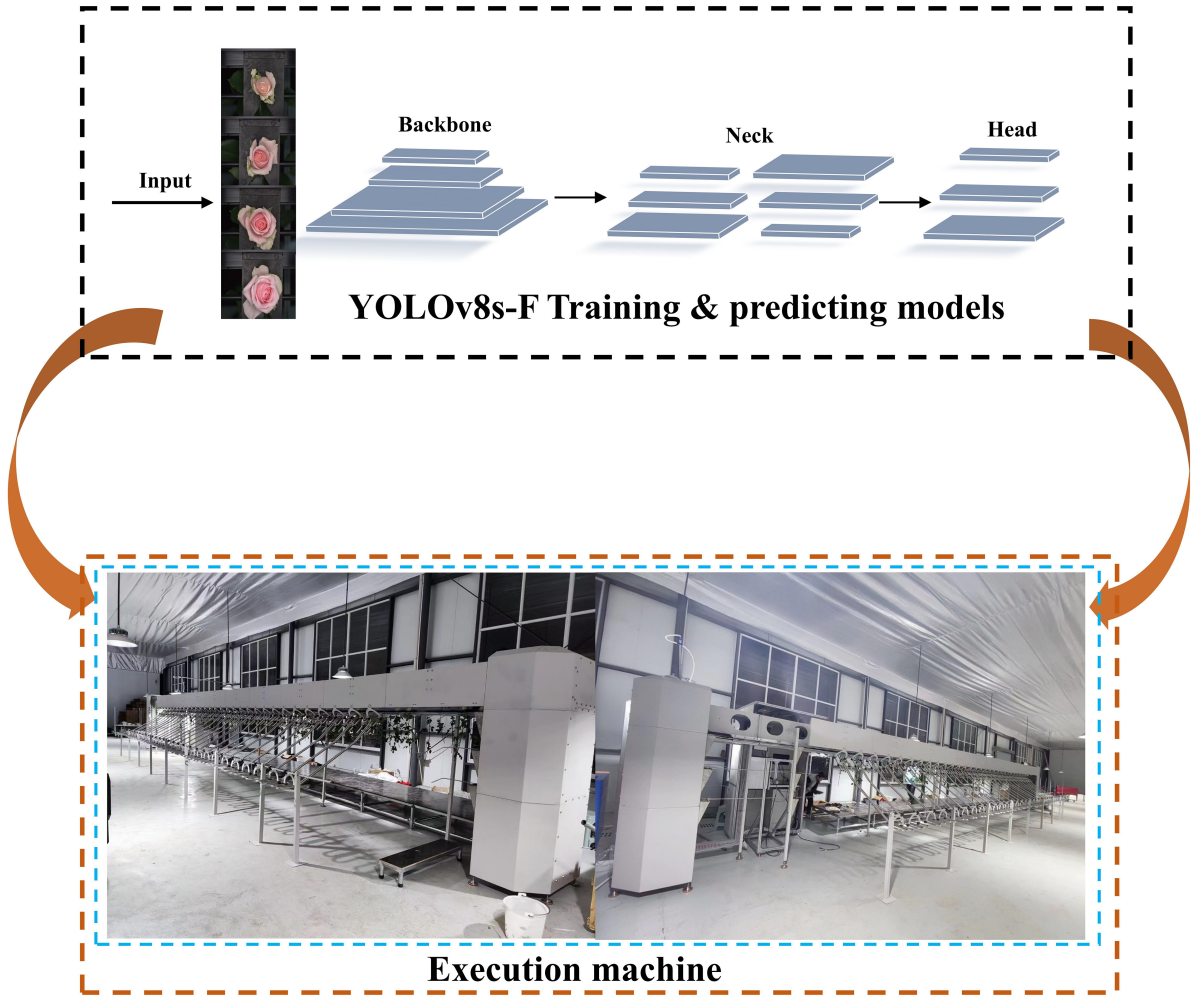
Field experiment.

**Figure 8:**
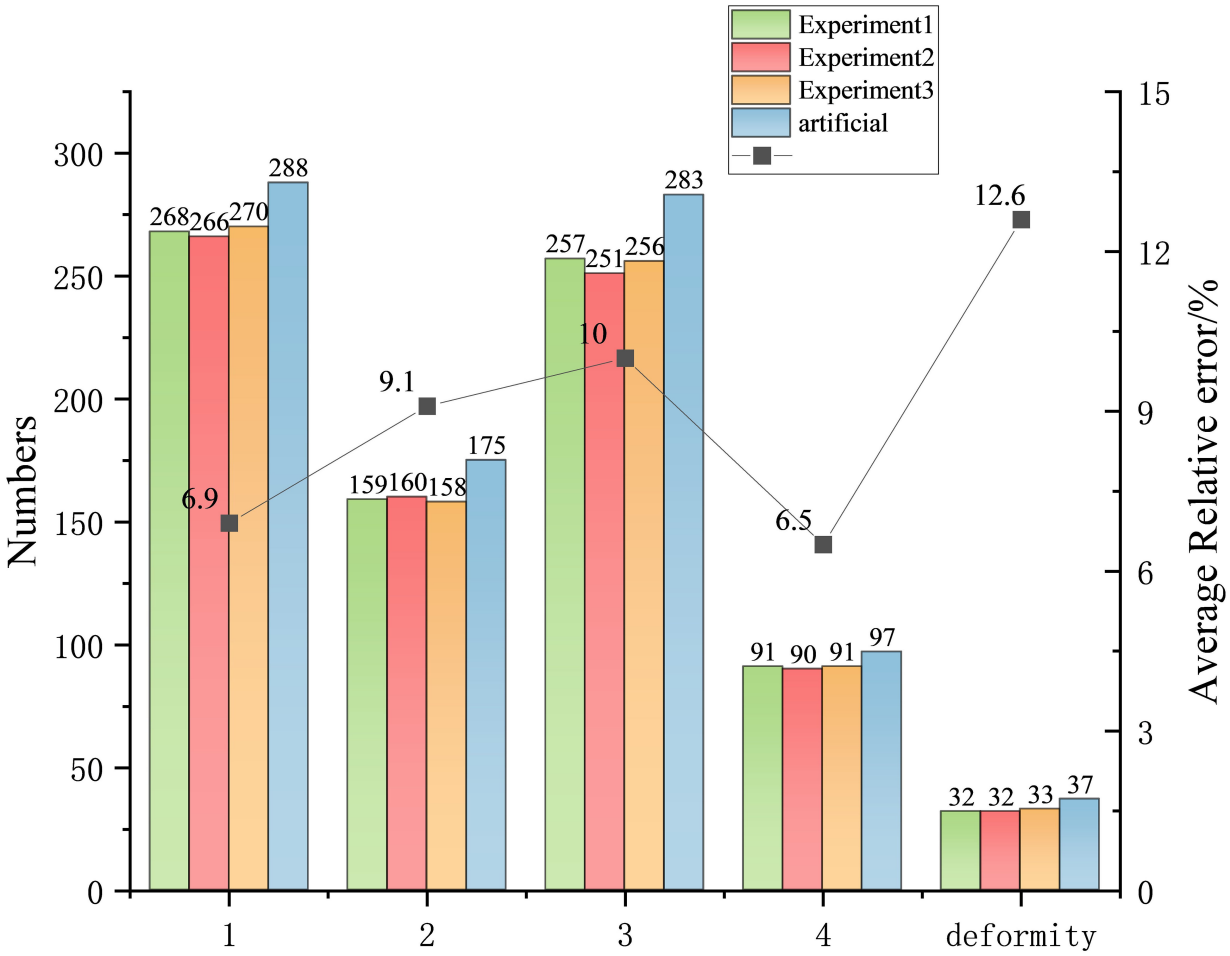
Field experiment results.

In February 2024, we conducted a field experiment on the grading of the openness of fresh-cut rose flowers at the National Flower Seedling Base in Luxi County, Honghe Autonomous Prefecture, Yunnan Province, China, as illustrated in **Figure 7**. A total of 813 images of fresh-cut roses were randomly collected, including White Avalanche, Sweet Avalanche, and Peach Avalanche. The experimental data is presented in **Figure 8**. After identification by on-site technicians, the quantities of flowers classified as degree 1, degree 2, degree 3, degree 4, and deformed flowers were 288, 175, 283, 97, and 37, respectively. Consequently, three experiments were conducted for each category, calculating the relative error between the algorithm’s predicted results and the ground truth values. The average relative error for each category was computed to one decimal place.

The experimental results show that the relative errors for degrees 1, 2, 3, and 4 and the deformed flowers were 6.9%, 9.1%, 10.0%, 6.5%, and 12.6%, respectively, as illustrated in **Figure 8**. The algorithm performed best in detecting the degree 4 category and worst in identifying the deformed flowers. The recognition of degree 2 and 3 flowers had relatively large errors with similar values. Overall, the YOLOv8s-F model can meet the grading requirements of the automatic grading machine for fresh-cut roses.

## 4 Discussion

**Figure 9:**
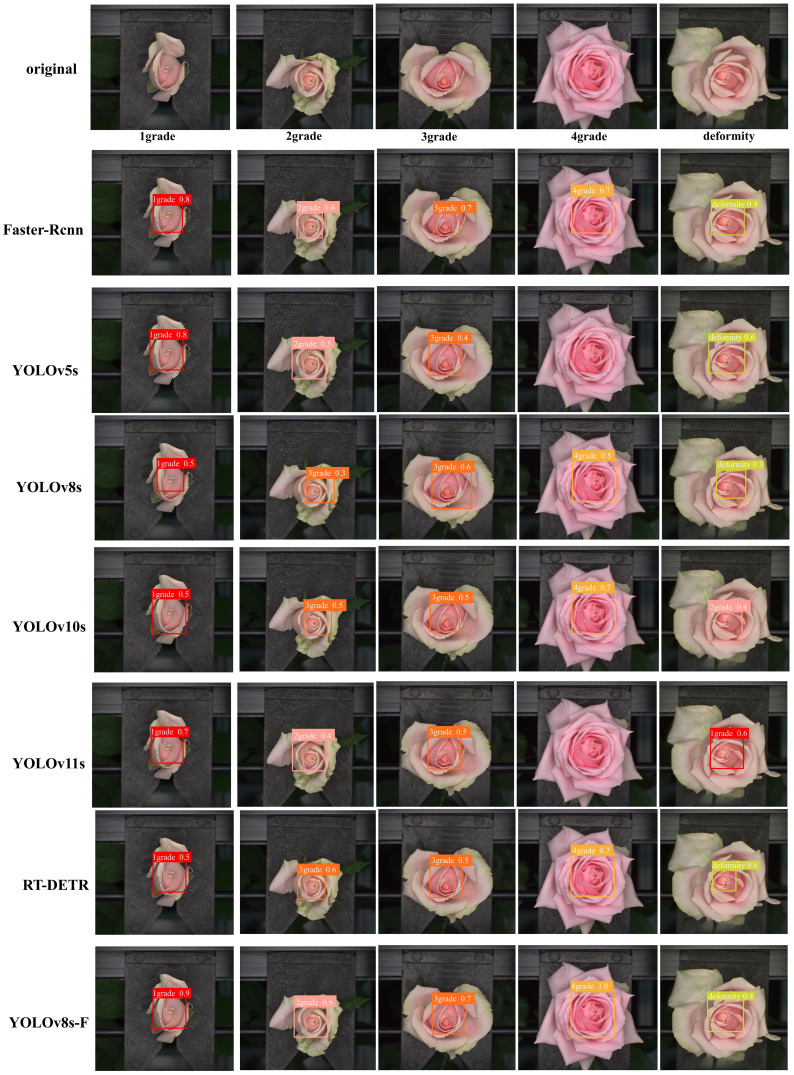
Comparison of the detection results between YOLOv8s-F and other models.

**Figure 9** illustrates the detection performance of YOLOv8s-F and other models on the same data. YOLOV8s-F and Faster R-cnn accurately detect the positions of the unopened inner petals and predict their categories. YOLOv11s and YOLOv5s have missed detections for degree 4. YOLOv11s and YOLOv10s has misclassified the deformed flowers. RT-DETR andYOLOv8s and YOLOv10s has misclassified the degree 2 flowers. Faster Rcnn achieved detection results similar to YOLOV8s-F due to its two-stage algorithm and larger model. However, due to larger models, larger FLOPs, and slower detection speeds, the deployment potential of Faster Rcnn on classifiers is inferior to our model. In the end-to-end model, there are varying degrees of false detections of second degree flowers, including missed detections of fourth degree flowers, and false detections of deformity flowers. The YOLOV8s-F model proposed in this study ensures the quality of feature extraction while reducing the complexity of the lightweight backbone network MobileNetV3. The addition of EMA attention mechanism in the feature fusion stage enhances the model's attention to advanced semantic features, and Wise-IoU improves the attention to more difficult categories in model detection and classification. So YOLOV8s-F shows better performance than other end-to-end models when facing second and third degree flowers with similar external features, fourth degree flowers with fully unfolded inner petals, and deformity flowers with unopened inner petals.

Through the analysis of on-site experimental results, it was found that the model has a relatively large relative error in grading degree 2 and 3 and malformed flowers compared to manual prediction, and the relative error values are comparable. The looseness of the inner petals of degree 2 and 3 fresh-cut flowers is greater in summer, leading to differences in the opening of the inner petals for flowers detected in field experiments. However, the model pays more attention to the inner petals, resulting in misclassification of the model. In the future, we will continue to expand the dataset seasonally to address potential data imbalance issues, improve the model’s generalization ability, and reduce the relative errors with manual predictions. Given the small number of malformed flowers in the dataset compared to other categories, the model’s classification accuracy for malformed flowers is not high. This is also clear in the map values of malformed flowers in **Table 4**. Therefore, we will continue to collect images of malformed flowers to expand the dataset and improve the algorithm’s ability to recognize this type of flower. Meanwhile, the model architecture will be adjusted to adapt to larger-scale data training while ensuring high computational efficiency and detection speed. Moreover, we will expand the dataset with more rose varieties so that we will be able to grade the majority of fresh-cut roses on the market for openness. In addition, an automatic flower-loading mechanism will be added to the grading machine to achieve fully automatic grading. Finally, the speed of the classifier operation and experiment will be accelerated to verify the performance of the vision system and prepare for the improvement of production efficiency.

## 5 Conclusion

This study proposes an improved YOLOv8s network model, YOLOv8s-F, for the classification of the openness of fresh-cut roses. The core technical contributions are as follows:

1. The backbone network of YOLOv8s is replaced with MobileNetV3, introducing the EMA attention mechanism module in the Neck part and adopting the Wise-IoU loss function. These improvements to YOLOv8s increase the model’s map@0.5 (%) by 4.5% and the precision by 11% while reducing FLOPs by 16.5% and the model size by 30%.
2. YOLOv8s-F outperforms SSD, Faster R-CNN, YOLOV3, YOLOV5s, YOLOV7, YOLOV8s, RT-DETR, YOLOV10 and YOLOV11 in terms of comprehensive performance. It achieves the best performance balance in comparison with Faster R-CNN, and it is more suitable for the classification of the openness of fresh-cut roses. When comparing the EMA attention mechanism to CA, CBAM, SE, and ECA, EMA generates the highest map@0.5 (%).
3. YOLOv8s-F is applied to the self-developed automatic grader for fresh-cut roses and conducted onsite experiments. The results highlight that the relative errors between the grading results and manual predictions are 6.9%, 9.1%, 10.0%, 6.5% and 12.6% for the five categories of flowers (degrees 1, 2, 3, and 4 and malformed), indicating that YOLOv8s-F performs well in practical applications.

In conclusion, the improved model YOLOv8s-F can meet the requirements for the detection of the openness of fresh-cut roses, providing technical support for the visual detection system of the automatic grader for fresh-cut roses.

## Data Availability

Source code and datasets can be obtained by contacting the corresponding author. Requests to access the datasets should be directed to yzzzzww@163.com.
